# Honoring Cultural Strength: Navigating Formal & Informal Mutual Aid Among Grandparent Caregivers

**DOI:** 10.1093/geroni/igaf122.1170

**Published:** 2025-12-31

**Authors:** Gaynell Simpson, Wendy Lustbader

## Abstract

Symposium Overview: The formal and informal mutual aid of grandparent caregivers are often overlooked or underutilized, yet they serve as vital sources of support. This symposium highlights the cultural assets that African American and Latina communities have long accessed as sources of strength. Historically, underserved grandparents have leaned on mutual aid practices such as neighbors relying on each other, sharing produce from community gardens, exchanging skills and abilities, and standing together with extended families-especially when societal resources were inaccessible. Today, these networks are more critical than ever. With families becoming more geographically dispersed and formal support systems strained by reductions in government assistance, the role of community-based mutual aid has only grown in significance. Grandparents raising grandchildren must navigate these evolving challenges while drawing on deep-rooted cultural traditions of collective caregiving. Four researchers and practitioners with expertise within these communities will present insights and practical recommendations that can make a difference, informing both researchers and practitioners committed to assisting them in contemporary times. Carole Cox presents a virtual empowerment program focused on skill building, social connection and empowerment. Nancy Mendoza offers practical recommendations on how the cultural assets of Latino grandparent caregivers can enhance their caregiving experience. Tina Peterson examines mutual aid among African American grandmothers raising grandchildren, highlighting how they compensate for gaps in formal and informal services through support networks. Gaynell Simpson highlights the need for intergenerational programs and macro-level advocacy to address systematic inequalities impacting grandparent caregivers and their families.

